# Postoperative Refraction in the Second Eye Having Cataract Surgery

**DOI:** 10.5402/2011/273923

**Published:** 2011-12-10

**Authors:** Christopher T. Leffler, Martin Wilkes, Juliana Reeves, Muneera A. Mahmood

**Affiliations:** ^1^Eye Clinic 112, Ophthalmology Section, Hunter Holmes McGuire VA Medical Center, 1201 Broad Rock Boulevard, Richmond, VA 23249, USA; ^2^Department of Ophthalmology, Virginia Commonwealth University School of Medicine, P.O. Box 980438, 401 North 11th Street, Richmond, VA 23298, USA; ^3^Cincinnati Eye Institute, Cincinnati, OH 45242, USA

## Abstract

*Introduction*. Previous cataract surgery studies assumed that first-eye predicted and observed postoperative refractions are equally important for predicting second-eye postoperative refraction. *Methods*. In a retrospective analysis of 173 patients having bilateral sequential phacoemulsification, multivariable linear regression was used to predict the second-eye postoperative refraction based on refractions predicted by the SRK-T formula for both eyes, the first-eye postoperative refraction, and the difference in IOL selected between eyes. *Results*. The first-eye observed postoperative refraction was an independent predictor of the second eye postoperative refraction (*P* < 0.001) and was weighted more heavily than the first-eye predicted refraction. Compared with the SRK-T formula, this model reduced the root-mean-squared (RMS) error of the predicted refraction by 11.3%. *Conclusions*. The first-eye postoperative refraction is an independent predictor of the second-eye postoperative refraction. The first-eye predicted refraction is less important. These findings may be due to interocular symmetry.

## 1. Introduction

Cataract surgeons strive to achieve the desired postoperative refraction by predictive algorithms which enable optimal intraocular lens (IOL) selection [1–12]. Inaccuracies in prediction of postoperative refraction might occur because of errors in parameters which are measured (axial length and anterior corneal curvature) [1, 11] and also due to inaccurate assumptions about parameters which are not typically analyzed or measured preoperatively, such as postoperative anterior chamber depth [3, 11], posterior corneal curvature, corneal thickness and asphericity, pupil size, and tissue refractive indices [11]. One analysis indicated that measurement errors in axial length and keratometry account for 19% of the error in postoperative refraction, other anatomic variables account for 52%, IOL tolerances account for 1%, and errors in refraction account for 27% [11]. The analysis explicitly did not address axial chromatic aberration [11], which is on average 2.5 diopters over visible wavelengths (400–700 nm) [13].

Recent work has addressed whether the information from the first eye sheds light on the expected postoperative refraction of the second eye having LASIK [14] or cataract surgery [4–8, 12]. Several approaches might be taken. Measured biometric variables (axial length [4] and keratometry [4, 15]) are highly correlated between eyes. Therefore, one might assume that the axial length and keratometry values of the second eye are similar to those of the first eye. For instance, if the first-eye axial length is 23.5 mm and the second-eye axial length is 23.3 mm, one might suspect that the axial length measurement in the second eye underestimates the true value. By this logic, the greater axial length in the first eye would lead to selection of a *lower*-power intraocular lens in the second eye.

An alternate approach is to assume that the error in refraction of the first eye (observed minus expected postoperative refraction) will be observed (in whole or in part) in the second eye [4–8]. This approach implicitly recognizes that the eyes might be similar in ways not directly measured preoperatively. For instance, Myrowitz et al. found that central corneal thickness was highly correlated between eyes [15]. Jabbour et al. found that adjusting the IOL power in the second eye by the error observed in the first eye did not improve prediction accuracy [4]. However, in more recent analyses which were more flexible (i.e., had an additional degree of freedom because they allowed the error to be scaled), correcting for half [5, 8], or nearly half [7], the error in the first eye refractive prediction did improve the second eye refractive prediction. By this logic, when the first-eye axial length is greater, a more myopic prediction for this eye is made, the subsequent refraction is interpreted as a more hyperopic (or less myopic) error, and a *higher* power intraocular lens is selected for the second eye.

The present work sought to clarify these approaches and extend previous analyses by using multivariable regression to determine how the biometric and refractive information from the first eye should be applied to the second eye.

## 2. Materials and Methods

The study was carried out after approval by the institutional review board of the McGuire VA Medical Center. Cataract surgeries over a 65-month period from 2004 to 2009 at the center were reviewed. Exclusion criteria were previous ophthalmic surgery on the studied eye, complications during the surgery preventing placement of an intraocular lens in the capsular bag, placement of a lens other than the AcrySof SA60AT lens (Alcon Laboratories), postoperative macular edema, and postoperative best corrected visual acuity of less than 20/60.

All patients had a preoperative evaluation including best corrected visual acuity using the Snellen chart, refraction by retinoscopy with subjective refinement, slit lamp, and dilated funduscopic examination.

Prior to 2007, axial length measurements were performed by an ophthalmic technician using the I^3^ SYSTEM ABD-v2 (Innovative Imaging Inc. Sacramento, CA, USA) immersion A-scan. Keratometry measurements were performed using a Bausch & Lomb keratometer.

Beginning in 2007, new patients had axial length and keratometry measurements performed with the IOL-Master system (Carl Zeiss Meditec) if clarity of the optical media permitted. For each patient, the IOL power was then selected by the surgeon using the SRK/T formula [1], which functions as well as other commonly used equations [16].

All patients underwent phacoemulsification with placement of the AcrySof SA60AT lens in the bag (patients with other lenses were excluded). Routine postoperative examination and followup included visits at 1 day, 1-2 weeks, and 6–8 weeks postoperatively. Postoperative refraction by retinoscopy with subjective refinement and best corrected visual acuity were recorded at 6 to 8 weeks following the surgery.

### 2.1. Statistical Evaluation

The optimal lens A constants for the immersion ultrasound group and for the IOL-master group were determined (in separate analyses) by iteratively determining the A constants which minimized the mean absolute error (MAE) of each group. The manufacturer's nominal A constant does not apply to all biometry methods, such as the IOL-Master. Moreover, by “personalizing” the A constants for our practice, we are optimizing the SRK-T predictions for the dataset. Therefore, any improvements made possible by incorporation of first-eye information are not due to lack of optimization of the SRK-T prediction. Predictors of the observed postoperative refraction in the second eye having cataract surgery (*R*_2*O*_) were determined by univariate and multivariable linear regression analysis. Independent variables included: predicted postoperative refraction in first (*R*_1*P*_) and second (*R*_2*P*_) eyes by the SRK-T formula, observed postoperative refraction in the first operated eye (*R*_1*O*_), and difference in IOL powers between eyes (*P*_2_ − *P*_1_). Model performance was evaluated by the MAE, and by the root-mean-squared (RMS) error, which is appropriate for evaluating standard least squares regression, which minimizes the squared error [12]. For analyses incorporating biometric variables, separate analyses for the IOL-Master and ultrasound eyes were performed. Statistica version 7 software (StatSoft, Inc., Tulsa, OK, USA) was used to generate the regression coefficient (b), coefficient of determination (*r* ^2^), and statistical significance (*P* values).

## 3. Results

One hundred seventy-three patients had bilateral phacoemulsification and met the inclusion criteria. The median age was 73 years (interquartile range 18 years), 98% were male, 74% were white, and 25% were black. Age and preoperative biometry values are described in Table 1. One hundred eight patients had biometry with immersion ultrasound in both eyes, 60 patients had biometry with the IOL-Master in both eyes, and 5 patients had immersion ultrasound used for the first eye but the IOL-Master for the second eye. Ninety-five per cent of the 173 patients had an absolute interocular difference of mean keratometry of 0.88 D or less, and 95% of the patients had an absolute interocular difference of axial length of 0.45 mm or less.

The A constant value of 118.5 was optimal in minimizing the MAE (0.528 D) for the eyes measured with ultrasound (*n* = 221). The A constant value of 118.8 was optimal in minimizing the MAE (0.504 D) for the eyes measured with the IOL-Master (*n* = 125). These values are similar to the MAE values of 0.63 D [4] and 0.44 to 0.47 D [5] observed previously with contact ultrasound biometry.

**Figure 1:**
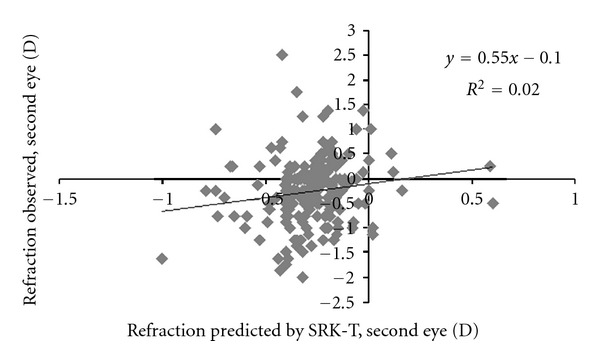
Prediction of postoperative refraction in the second eye by the SRK-T formula.

The conventional biometric prediction model (SRK-T) predicted 2% of the variation in the postoperative refraction in the second eye (Table 2, Figure 1). The strongest single predictor of the postoperative refraction in the second eye was the postoperative refraction in the first eye (denoted *R*_1*O*_, *r* ^2^ = 0.21, *P* < 0.001, Table 2). In fact, the first-eye refraction *R*_1*O*_ was the only significant independent predictor of *R*_2*O*_ in the multivariable analysis (Table 2, *P* < 0.001). The complete multivariable model was

(1) $$\begin{array}{cc} R_{2O} & =-0.09+0.44R_{1O}+0.43R_{2P}-0.09R_{1P}\\ & \;-0.07\left(P_{2}-P_{1}\right). \end{array}$$

The absolute value of the regression coefficient for the first-eye refraction (0.44) was much larger than that of the coefficient for the first-eye predicted refraction (−0.09), suggesting that the first-eye biometry (axial length and keratometry) was of lesser importance. The complete model can be rewritten as the sum of two approaches to prediction:

(2) $$\begin{array}{cc} R_{2O} & =-0.09+0.43\;\left[R_{2P}+0.21\left(R_{1O}-R_{1P}\right)\right]\\ & \;+0.35\left[R_{1O}-0.2\left(P_{2}-P_{1}\right)\right], \end{array}$$

where [*R*_2*P*_ + 0.21(*R*_1*O*_ − *R*_1*P*_)] represents the standard SRK-T prediction plus the scaled error in the first eye refraction (similar to previous models [4, 5, 7, 8]), and the [*R*_1*O*_ − 0.2(*P*_2_ − *P*_1_)] term constitutes a “refraction-only” or “biometry-independent” portion of the model.

**Figure 2:**
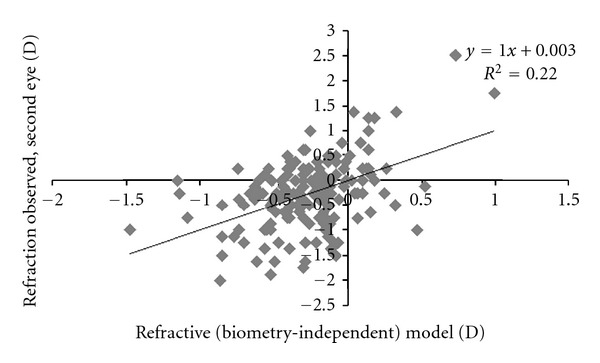
Prediction of postoperative refraction in the second eye (*R*_2*O*_) by the refractive (biometry-independent) model: *R*_2*O*_ = 0.44*R*_1*O*_ − 0.08(*P*_2_ − *P*_1_) − 0.19, where *R*_1*O*_ is refraction observed postoperatively in the first eye and (*P*_2_ − *P*_1_) is change in IOL power between eyes.

Indeed, a multivariable model can be constructed based solely on refraction (without including any biometric variables):

(3) $$R_{2O}=-0.19+0.44R_{1O}-0.08\left(P_{2}-P_{1}\right),$$

where *P* < 0.001 for *R*_1*O*_, *P* = 0.14 for (*P*_2_ − *P*_1_), and the model *r* ^2^ = 0.22 (Figure 2).

Previous investigators [4, 5, 7, 8] used the predictors of *R*_2*P*_ and *R*_1*O*_ − *R*_1*P*_ (but not *P*_2_ − *P*_1_). Therefore, the following multivariable model was constructed (*r* ^2^ = 0.22):

(4) $$R_{2O}=-0.09+0.44R_{1O}+0.48R_{2P}-0.14R_{1P},$$

where *P* < 0.001 for *R*_1*O*_, *P* = 0.58 for *R*_1*P*_, and *P* = 0.06 for *R*_2*P*_.

Table 3 compares models not only by the coefficient of determination (*r* ^2^) but also by the mean absolute error (MAE) and root mean square (RMS) error values typical in postsurgery analyses. First, we confirmed that adding the first-eye error in postoperative refraction to the SRK-T prediction for the second eye increases the MAE [4] to 0.57 D, compared with 0.52 D for the SRK-T formula alone. In addition, we confirmed the finding that adding half the error in first-eye refraction decreased the MAE in the second eye [5, 8] (in our case to 0.50 D). The RMS error showed similar changes (Table 3). Using linear regression to optimally scale and offset the predictions decreased the MAE and RMS error for all models. Any scaled model which included the first-eye refraction (*R*_1*O*_) had a high *r* ^2^ ≥ 0.21 and had an improved MAE of between 0.48 and 0.49 D (Table 3). The complete model reduced the root-mean-squared (RMS) error of the predicted refraction by 11.3%, while a model based solely on refraction (without biometric variables) reduced the RMS error by 10.5%. 

### 3.1. Subgroup Analysis

Similar findings were seen in subgroup analysis. In the 60 patients for whom the IOL-Master was used for both eyes, the standard SRK-T equation (*R*_2*P*_) predicted a similar degree of variance in postoperative second-eye refraction (*r* ^2^ = 0.025, *P* = 0.22 for the *R*_2*P*_ term in univariable analysis). The multivariable model in these patients was (*r* ^2^ = 0.24) as follows:

(5) $$\begin{array}{cc} R_{2O} & =-0.12+0.46R_{1O}+0.37R_{2P}-0.20R_{1P}\\ & \;-0.07\left(P_{2}-P_{1}\right). \end{array}$$

The only significant term was the first eye refraction (for *R*_1*O*_, *P* < 0.001, all other *P* > 0.4).

In the 108 patients having immersion ultrasound and manual keratometry for both eyes, the standard SRK-T equation (*R*_2*P*_) predicted a similar degree of variance in postoperative second-eye refraction (*r* ^2^ = 0.01, *P* = 0.32 for *R*_2*P*_ term in univariable analysis). The multivariable model in these patients was (*r* ^2^ = 0.22) as follows:

(6) $$\begin{array}{cc} R_{2O} & =-0.13+0.44R_{1O}+0.29R_{2P}-0.08R_{1P}\\ & \;-0.06\left(P_{2}-P_{1}\right). \end{array}$$

The only significant term was the first-eye refraction (*P* < 0.001, all other *P* > 0.35).

## 4. Discussion

This study determined that the postoperative refraction in the first operated eye is an independent predictor of the refraction after cataract surgery in the second eye. It is striking that a refraction-only model, completely free of biometric information, performed better than standard biometric models. (Of course, the best model used both biometry and first-eye refraction, and both types of information should be considered in clinical practice.)

The standard SRK-T prediction model for the second eye had a regression coefficient similar in magnitude, and performed as well as in previous studies, based on the mean absolute error. The SRK-T prediction model and biometry of the first operated eye were of lesser importance. If confirmed, the regression equations could be used to predict refraction after second-eye surgery.

Previous analyses [4, 5, 7, 8] assumed that the first-eye predicted and observed postoperative refractions were equal in importance. In our analysis, these terms were scaled independently. The first-eye predicted refraction, which turned out to be much less important, is based on biometric measurements and on the selected IOL power. We demonstrated in the introduction that consideration of interocular symmetry in measured parameters and consideration of symmetry in unmeasured parameters have opposite effects on second-eye IOL selection. If first and second eyes are identical in ways both measured and unmeasured, then placement of the same lens in the second eye would result in the same refraction as was observed in the first eye. Opposing effects make the first eye biometry irrelevant. This principle is demonstrated in mathematical terms in the appendix.

Revisiting a hypothetical case [5] illustrates how the method might be applied. The first eye is implanted with a lens of power *P*_1_, is predicted by the SRK-T formula to have a refraction of *R*_1*P*_ = −0.02 D, but is observed to have a postoperative refraction of *R*_1*O*_ = 1.0 D. If placing a lens with power *P*_2_ in the second eye is predicted by the SRK-T formula to produce a refraction of *R*_2*P*_ = −0.76 D and if *P*_2_ − *P*_1_ = 1 in this hypothetical case, then one can plug *R*_2*P*_, *R*_1*O*_, *R*_1*P*_, and *P*_2_ − *P*_1_ into (1) to calculate the second eye refraction of *R*_2*O*_ = −0.05 D.

This study also confirmed the effect that addition of the first eye error in refraction has on the mean absolute error of the second eye. Specifically, addition of the full error worsens the prediction [4], while addition of half the error improves the prediction [5, 8]. The utility of first-eye refraction was observed despite the fact that the traditional biometry model was as accurate as in previous experiences, as detailed in Section 3. One objection to consideration of the first eye refraction is that only incremental improvements are seen. For instance, the complete model reduced the root-mean-squared (RMS) error of the predicted refraction by 11.3%—an improvement similar to that seen for astigmatism [12]. On the other hand, such improvements can have a major public health impact when extrapolated to the millions of cataract surgeries performed annually. Moreover, the cost is minimal, because refraction of the first eye is already performed and requires no capital investments in new technologies.

The findings may be specific to our particular practice: mostly male patients and multiple surgeons in a teaching environment. In addition, the findings are based on retrospective calculations. As 95% of the patients had an interocular difference in mean keratometry readings of less than 0.88 D and in axial length of less than 0.45 mm, the method should not be applied for patients with a greater degree of interocular asymmetry, until additional data are available. The strongest evidence would come from a large, prospective trial randomizing patients to consideration of first-eye refraction or standard care. Our finding that the first-eye refraction is weighted more heavily than first-eye biometry measurements, as opposed to the equal weighting previously assumed, can help in designing such a trial. This study suggests that consideration should be given to delaying second-eye surgery until an accurate postoperative refraction can be obtained in the first eye.

## 5. Conclusions

The first eye postoperative refraction is an independent predictor of the second eye postoperative refraction. In contrast with previous assumptions, the first-eye predicted refraction is a less important predictor. These findings may be due to interocular symmetry in both measured and unmeasured variables. The regression equation prediction can be considered when selecting an intraocular lens for the second eye in patients who meet the above criteria for interocular symmetry of axial length and keratometry.

## Figures and Tables

**Table 1:** Patient age and preoperative biometry in the second operative eye.

	Mean (SD)	Range
Age (years)	71.2 (9.9)	45–89
Keratometry, manual (D, *n* = 108)	43.3 (1.4)	40.0–47.1
Axial length, ultrasound (mm, *n* = 108)	23.6 (0.80)	21.1–25.6
Keratometry, IOL-Master (D, *n* = 65)	44.0 (1.3)	41.9–47.1
Axial length, IOL-Master (mm, *n* = 65)	23.6 (0.97)	21.1–26.3

**Table 2:** Prediction of refraction in the second eye by univariate and multivariable analysis.

Variable	Univariate analysis				Multivariable model (*r* ^2^ = 0.23)	
	Intercept	b*	*P*	*r* ^2^	b*	*P*
*First eye*						
Observed refraction (*R*_1*O*_)	−0.19	0.44	<0.001	0.21	0.44	<0.001
Predicted refraction (*R*_1*P*_)^†^	−0.15	0.41	0.19	0.01	−0.09	0.71
*Second eye*						
Predicted refraction (*R*_2*P*_)^†^	−0.10	0.55	0.04	0.02	0.43	0.09
Difference in lens power (*P*_2_ − *P*_1_)	−0.25	−0.08	0.18	0.01	−0.07	0.24
Intercept (multivariable model).	—	—	—	—	−0.09	0.32

*b: regression coefficient.

^†^Predicted refraction in first (*R*_1*P*_) and second (*R*_2*P*_) eyes based on SRK-T formula.

**Table 3:** Comparison of model performance.

Model name	Definition*: *R*_2*O*_ =	MAE (D)^†^	RMS (D)^‡^	*r* ^2^
SRK-T	*R*_2*P*_	0.518	0.689	—
SRK-T with scaling	0.55*R*_2*P*_ − 0.10	0.513	0.683	0.02
Full error offset [4]	*R*_2*P*_ + (*R*_1*O*_ − *R*_1*P*_)	0.573	0.734	—
Full error offset with scaling	0.45[*R*_2*P*_ + (*R*_1*O*_ − *R*_1*P*_)] − 0.18	0.488	0.612	0.22
50% error offset [5, 8]	*R*_2*P*_ + 0.5(*R*_1*O*_ − *R*_1*P*_)	0.497	0.620	—
50% error offset with scaling	0.80[*R*_2*P*_ + 0.5(*R*_1*O*_ − *R*_1*P*_)] − 0.07	0.489	0.614	0.21
Refraction only	0.44*R*_1*O*_ − 0.08(*P*_2_ − *P*_1_) − 0.19	0.485	0.611	0.22
Prior terms	0.44*R*_1*O*_ + 0.48*R*_2*P*_ − 0.14*R*_1*P*_ − 0.09	0.489	0.608	0.22
Complete multivariable	0.44*R*_1*O*_ + 0.43*R*_2*P*_ − 0.09*R*_1*P*_ − 0.07(*P*_2_ − *P*_1_) − 0.09	0.487	0.606	0.23

*Observed postoperative refraction in first (*R*_1*O*_) and second (*R*_2*O*_) eyes. Predicted refraction in first (*R*_1*P*_) and second (*R*_2*P*_) eyes based on SRK-T formula. *P*_2_ − *P*_1_: difference in intraocular lens powers for first and second eyes.

^†^MAE: mean absolute error (diopters).

^‡^RMS: root-mean-squared error (diopters).

## Appendix

The SRK-1 formula is no longer used in practice, but its simplicity helps in illustrating why certain variables might be important [2]. A variant of this formula is

(A.1) $$\begin{array}{c} P_{nE}=A-2.5L_{n}-0.9K_{n},\\ R_{nP}=\frac{\left(P_{nE}-P_{n}\right)}{c}, \end{array}$$

where *P*_*nE*_: power of lens expected to produce emmetropia in eye *n*, *L*_*n*_: axial length of eye *n* (mm), *K*_*n*_: mean corneal curvature by keratometry in eye *n* (D), *A*: *A*-constant, specific for each intraocular lens and biometry system, *R*_*nP*_: predicted postoperative refraction of eye *n* (D), *P*_*n*_: power of lens selected for eye *n* (D), and *c*: dimensionless constant (1 for *P* ≪ 14, 1.25 for *P* ≫ 14).

The observed refraction is

(A.2) $$R_{nO}=R_{nP}+E_{n},$$

where *E*_*n*_ represents the unexpected error in refraction in eye *n*.

Current biometry formulas, such as the SRK-T equation, are still based on parameters subject to measurement error, such as axial length (*L*) and keratometry (*K*). Because of interocular symmetry, the first-eye measurement provides information about the second eye. In the limiting case that the eyes are identical in length and curvature, the best estimate comes from averaging the values. For the second eye, the predicted refraction is

(A.3) $$R_{2P}=\frac{\left(\left(P_{1E}\;+\;P_{2E}\right)/2-P_{2}\right)}{c}.$$

When the eyes are similar in ways that are measured, as *P*_1*E*_ decreases (because the first eye keratometry *K*_1_ or axial length *L*_1_ increase), one expects a more myopic refraction in the second eye.

On the other hand, refraction is affected by quantities which are not analyzed, such as final axial position of intraocular lens within the eye [11]. These factors are reflected in the unexplained error term, *E*_*n*_. Previous models [5, 7, 8] assumed that the errors between the two eyes are related by *E*_2_ = *f*∗*E*_1_, where *f* = 1 [4], 0.5 [5, 8], or varied between 0.27 and 0.56 (Olsen [7]) as follows:

(A.4) $$\begin{array}{c} E_{1}=R_{1O}-R_{1P}=R_{1O}-\frac{\left(P_{1E}-P_{1}\right)}{c},\\ E_{2}=f*E_{1}=f*\left(\frac{R_{1O}-\left(P_{1E}-P_{1}\right)}{c}\right),\\ R_{2O}=R_{2P}+E_{2},\\ R_{2O}=\frac{\left(P_{2E}-P_{2}\right)}{c}+E_{2},\\ R_{2O}=\frac{\left(P_{2E}-P_{2}\right)}{c}+f*\left(\frac{R_{1O}-\left(P_{1E}-P_{1}\right)}{c}\right). \end{array}$$

With this assumption, as *P*_1*E*_ decreases (because *K*_1_ or *L*_1_ increase), a more hyperopic refraction is expected in the second eye.

Thus, *P*_1*E*_, *L*_1_, and *K*_1_ can be either positively or negatively associated with *R*_2*O*_. Presumably, the eyes are similar in ways both measured and unmeasured, and both expressions can be combined.

If *f* = 1, so that *E*_2_ = *E*_1_, then

(A.5) $$\begin{array}{cc} R_{2O} & =\frac{\left(\left(P_{1E}+P_{2E}\right)/2-P_{2}\right)}{c}\\ & \;+\left(R_{1O}-\frac{\left(\left(P_{1E}+P_{2E}\right)/2-P_{1}\right)}{c}\right)\\ R_{2O} & =R_{1O}-\frac{\left(P_{2}-P_{1}\right)}{c}. \end{array}$$

This equation takes the form of (3) in the text, which fit the data well (Figure 2). With high degrees of interocular symmetry, then biometric values are irrelevant, and only the observed refraction in the first eye and the difference in IOL powers are important.
